# Maternal use of sedative drugs and its effects on pregnancy outcomes: a Finnish birth cohort study

**DOI:** 10.1038/s41598-021-84151-7

**Published:** 2021-02-24

**Authors:** Satu-Maarit Björkstedt, Hannu Kautiainen, Ulla Tuomi, Mika Gissler, Pirjo Pennanen, Johan G. Eriksson, Merja K. Laine

**Affiliations:** 1 Department of General Practice and Primary Health Care, University of Helsinki and Helsinki University Hospital, Helsinki, Finland; 2 Social Services and Health Care Division, City of Helsinki, Helsinki, Finland; 3 Folkhälsan Research Center, Helsinki, Finland; 4 Primary Health Care Unit, Kuopio University Hospital, Kuopio, Finland; 5 Pharmacy of Palokka, Jyväskylä, Finland; 6 Finnish Institute for Health and Welfare, Helsinki, Finland; 7 Karolinska Institute, Stockholm, Sweden; 8 Vantaa Health Center, Vantaa, Finland; 9 Department of Obstetrics and Gynecology and Human Potential Translational Research Programme, National University Singapore, Yong Loo Lin School of Medicine, Singapore, Singapore; 10 Agency for Science, Technology and Research (A*STAR), Singapore Institute for Clinical Sciences (SICS), Singapore, Singapore

**Keywords:** Disease prevention, Public health, Drug safety

## Abstract

Our aim was to evaluate maternal use of sedative drugs before, during, and after pregnancy and to assess the influence of use of these drugs on pregnancy outcomes. The study cohort (N = 6231) consists of all primiparous women, who lived in the city of Vantaa, Finland, and who delivered a singleton between 2009 and 2015. Data were obtained from Finnish national health registers. Of the women, 3.2% (n = 202) purchased at least once sedative drugs within 90 days before conception, during pregnancy and/or within 90 days after delivery. Sedative drug users were older, less likely to cohabitate, more often smokers, had lower educational attainment and had more mental diseases (for all p < 0.001) compared with non-users. Sedative drug users purchased more often antidepressants and drugs for the alimentary tract, musculoskeletal and nervous system than non-users (for all p < 0.001). No adverse birth or pregnancy outcomes were found in the group using sedative drugs compared with the non-users. Studies in larger cohorts are needed to confirm our study findings.

## Introduction

During the perinatal period up to 15% of women suffer from anxiety disorders and up to 50% from sleep disorders^1,2^. Sedative drugs such as benzodiazepines and benzodiazepine related drugs are generally prescribed for the treatment of anxiety and sleep disorders^3,4^. When treating women during the perinatal period, prescribing physicians have to balance between maternal wellbeing and potential harmful effects of the treatment on the fetus.

In the perinatal period, the prevalence of maternal use of sedative drugs varies between 1 and 14%, being lowest in Asia and highest in Eastern Europe^5^. A recent systemic review and meta-analysis reported that over the last decades there is no change in maternal use of sedative drugs^5^. However, a recent large Danish study reported a decrease in the prevalence of maternal use of sedative drugs, especially in women with psychiatric disease^6^. Primiparous women seem to suffer more anxiety and sleeping disorders than multiparous women, however, in some studies such a difference was not observed^7–10^. In pregnancy, sedative drugs cross the placenta with fetal uptake of the drug predisposing the neonate to harmful effects such as floppy infant syndrome, hypothermia, lethargy, respiratory problems, and withdrawal symptoms^11,12^. Further, maternal use of sedative drugs during pregnancy increases risk for spontaneous abortions, preterm births, caesarean sections, need for neonatal ventilatory support, and need for neonatal intensive care unit (NICU) treatment^13–16^. Previous studies have mainly evaluated maternal use of sedative drugs and pregnancy outcomes simultaneously in primiparous and multiparous women. To avoid the possible bias related to differences between primiparity and multiparity we studied primiparous women alone, something none of the previously published studies have done to the best of our knowledge.

Our aim was to evaluate maternal use of sedative drugs before, during, and after pregnancy and assess the influence of use of these drugs on pregnancy outcomes in primiparous women.

## Results

The mean age of the study participants (N = 6231) was 28.5 (standard deviation [SD] 5.2) years. Sedative drug users were older, more often living alone and smokers and had less years of schooling than non-users (for all p < 0.001). Mental diseases and epilepsy were more common in sedative drug users than in non-users (for mental diseases p < 0.001 and for epilepsy p = 0.017). Table 1 shows the characteristics of the primiparous women.

**Table 1:** Characteristics of the 6231 primiparous women divided into users of sedative drugs and non-users.

	Use of sedative drugs*		P-value
	No n = 6029	Yes n = 202	
Age (years), mean (SD)	28.5 (5.2)	29.8(5.8)	< 0.001
Cohabiting, n (%)	4831 (80)	141 (70)	< 0.001
**Smokers, n (%)**			< 0.001
No smoking	4938 (82)	148 (73)	
Quit during the first trimester	478 (8)	10 (5)	
Smoking during pregnancy	613 (10)	44 (22)	
Years of schooling, mean (SD)	13.6 (2.6)	12.8 (2.9)	< 0.001
Pre-pregnancy body mass index (kg/m^2^), mean (SD)	24.1 (4.6)	24.4 (4.8)	0.42
Obese (body mass index ≥ 30.0 kg/m^2^), n (%)	654 (11)	28 (14)	0.15
Morbidity, six most common chronic diseases**, n (%)			
Pulmonary diseases	184 (3)	8 (4)	0.46
Rheumatic diseases	77 (1)	0 (0)	0.18
Inflammatory bowel diseases	55 (1)	2 (1)	0.71
Mental diseases	44 (1)	12 (6)	< 0.001
Epilepsy	42 (1)	5 (2)	0.017
Diabetes mellitus	39 (1)	3 (1)	0.15
**Previous pregnancies (ectopic pregnancies, induced abortions, or miscarriages), n (%)**			0.031
None	4852 (80)	147 (73)	
One	832 (14)	37 (18)	
Two	257 (4)	15(7)	
Three or more	88 (1)	3 (1)	
Fertility treatment, n (%)	534 (9)	9(4)	0.029

*SD* standard deviation.

*Women who had prescription drug purchase with Anatomical Therapeutic Chemical (ATC) codes N03AE (clonazepam), N05BA (benzodiazepine derivatives anxiolytics), N05CD (benzodiazepine derivates), or N05CF (benzodiazepine related drugs) within 90 days before conception, during pregnancy, or within 90 days after delivery were considered as users of sedative drugs.

**Data on morbidity based on the register of reimbursement rights for chronic diseases kept by Social Insurance Institution, Finland.

### Purchases of prescription sedative drugs

**Figure 1:**
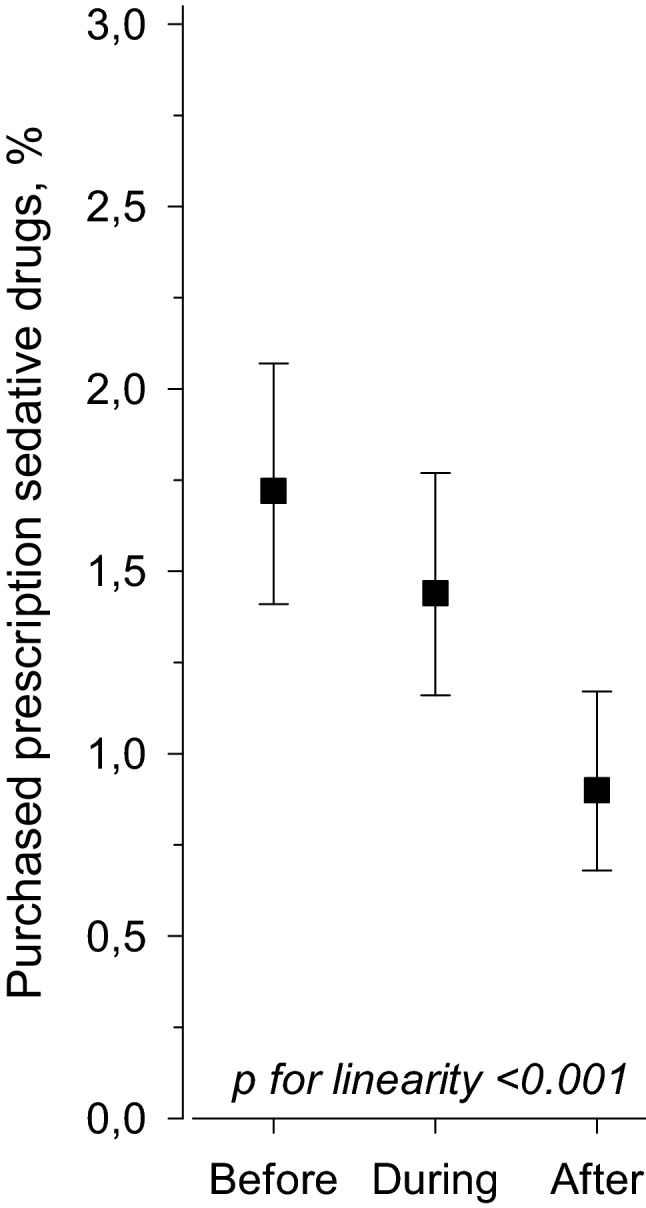
Percentage of the primiparous women who purchased prescription sedative drugs within 90 days before conception, during pregnancy or within 90 days after delivery. Prescription drug purchase with Anatomical Therapeutic Chemical (ATC) codes N03AE (clonazepam), N05BA (benzodiazepine derivatives anxiolytics), N05CD (benzodiazepine derivates), or N05CF (benzodiazepine related drugs) were considered as sedative drugs. Whiskers represents 95% confidence intervals.

Of the primiparous women, 3.2% (n = 202) purchased at least once sedative drugs within 90 days before conception, during pregnancy and/or within 90 days after delivery as prescribed by a physician. Of these 202 women, 125 purchased benzodiazepine or benzodiazepine related drugs. During pregnancy, 1.4% (n = 90) of the women purchased sedative drugs. Only six women purchased sedative drugs both within 90 days before conception, during pregnancy and within 90 days after delivery. The median number of sedative drug purchases was 1 (interquartile range [IQR] 1–2) within 90 days before conception, during pregnancy and within 90 days after delivery. Figure 1 shows the percentage of primiparous women who purchased sedative drugs within 90 days before conception, during pregnancy or within 90 days after delivery. The most commonly purchased prescription sedative drugs were benzodiazepine derivatives anxiolytics (Anatomical Therapeutic Chemical [ATC] code N05BA), 54.1% of all sedative drug purchases, and the second most benzodiazepine related drugs (ATC code N05CF), 41.6% of all sedative drug purchases.

### Purchases of prescription drugs

**Figure 2:**
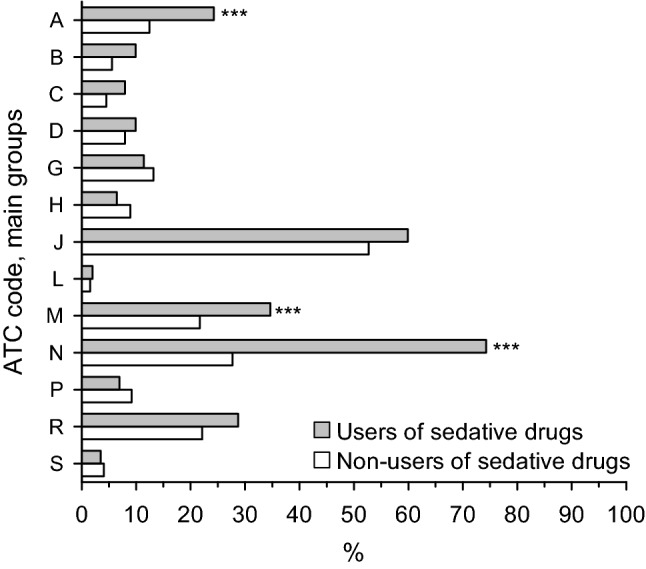
Proportion of primiparous women divided into sedative drug users and non-users who purchased some other prescription drugs than sedative drugs within 90 days before conception, during pregnancy or within 90 days after delivery. Prescription drug purchase with ATC codes N03AE (clonazepam), N05BA (benzodiazepine derivatives anxiolytics), N05CD (benzodiazepine derivates), or N05CF (benzodiazepine related drugs) were considered as sedative drugs. Hommel’s adjustment was applied to correct levels of significance for multiple testing: *p < 0.05, **p < 0.01, ***p < 0.001. *ATC* anatomical therapeutic chemical classification system codes, *A* alimentary tract and metabolism, *B* blood and blood forming organs, *C* cardiovascular system, *D* dermatological drugs, *G* Genitourinary system and reproductive hormones, *H* systemic hormonal preparations, excluding reproductive hormones and insulins, *J* anti-infectives for systemic use, *L* antineoplastic and immunomodulating agents, *M* musculoskeletal system, *N* nervous system excluding sedative drugs, *P* antiparasitic products, insecticides and repellents, *R* respiratory system, *S* sensory organs.

Women who purchased sedative drugs within 90 days before conception, during pregnancy or within 90 days after delivery also purchased more often all other prescription drugs (excluding sedative drugs) compared with non-users, 93.6% versus 78.5% (p < 0.001). Sedative drug users purchased more often antidepressants (drugs with ATC code N06A) than non-users, 56.9% versus 6.4% (p < 0.001). Figure 2 shows the proportion of primiparous women divided into sedative drug users and non-users who purchased some other prescription drugs than sedative drugs within 90 days before conception, during pregnancy or within 90 days after delivery.

### Use of sedative drugs and pregnancy outcomes

Table 2 shows pregnancy outcomes according to maternal use of sedatives drugs during pregnancy. No differences were observed in pregnancy outcomes between offspring of sedative drug users and non-users during pregnancy. Offspring of women who used sedative drugs during pregnancy had higher ponderal index compared with offspring of women without use of sedative drugs (p = 0.016).

**Table 2:** Pregnancy (N = 6231) and perinatal outcomes according to maternal purchases of prescription sedative drug during pregnancy.

	Maternal purchases of prescription sedative drug* during pregnancy		P-value
	None n = 6141	At least once n = 90	
Girls, n (%)	2954 (48.0)	41 (57)	0.11
Duration of pregnancy (weeks), mean (SD)	39.4 (2.0)	39.2 (2.2)	0.42
Preterm birth < 37 gestational weeks, n (%)	387 (56)	5 (6)	0.77
**Caesarean sections, n (%)**			0.042
Elective	315 (5)	10 (11)	
Urgent	947 (15)	18 (20)	
Emergency	92 (1)	1 (1)	
**Birth weight (g), mean (SD)**			
Boys	3471 (561)	3562 (481)	0.32
Girls	3369 (548)	3257 (690)	0.15
Birth weight (Z-score), mean (SD)	0.00 (1.00)	− 0.00 (1.11)	0.95
**Head circumference (cm), mean (SD)**			
Boys	35.1 (1.8)	35.2 (1.6)	0.80
Girls	34.6 (1.9)	34.0 (2.5)	0.052
Ponderal index (kg/m^3^), mean (SD)	27.4 (2.6)	28.1 (2.9)	0.016
Apgar score, 1 min, mean (SD)	8.4 (1.5)	8.5 (1.2)	0.64
**Before the age of seven days, n (%)**			
NICU treatment	695 (11)	16 (18)	0.056
Respirator treatment	105 (2)	2 (2)	0.67
Resuscitation with intubation	123 (2)	2 (2)	0.70
Antibiotic treatment	418 (7)	5 (6)	0.64
Diagnosis with ICD-10 codes Q00–Q99	610 (10)	7 (8)	0.50

*SD* standard deviation; *NICU* care at neonatal intensive care unit; *ICD-10 codes Q00–Q99* nternational Statistical Classification of Diseases and Related Health Problems, 10th revision, codes for congenital malformations, deformations and chromosomal abnormalities.

*Prescription drug purchase with Anatomical Therapeutic Chemical (ATC) codes N03AE (clonazepam), N05BA (benzodiazepine derivatives anxiolytics), N05CD (benzodiazepine derivates), or N05CF (benzodiazepine related drugs).

## Discussion

Of the primiparous women, 3% purchased at least once sedative drugs within 90 days before conception, during pregnancy and/or within 90 days after delivery. The most commonly purchased prescription sedative drugs were benzodiazepine derivatives anxiolytics. Sedative drug users more often purchased antidepressants than non-users. In offspring of sedative drug users there was not an increased need for NICU treatment during the first week of life.

According to a recent systematic review and meta-analysis, globally 2% of primiparous and multiparous women purchased benzodiazepines or benzodiazepine-related drugs within one year before conception, during pregnancy or within one year after delivery^5^. However, the variation between different regions was noteworthy: the highest prevalence was found in Eastern Europe (14%) and the lowest prevalence in Asia (0.9%), in North-western Europe the prevalence was 1.2%^5^. A Norwegian study including both primiparous and multiparous women reported that 2% of women purchased anxiolytics, hypnotics, and sedatives within 90 days before conception, almost 2% during pregnancy, and 1% within 90 days after pregnancy, respectively^17^. Our study findings of primiparous women, i.e. almost 2% purchased sedative drugs within 90 days before conception, almost 1.5% during pregnancy and almost 1% within 90 days after delivery, are in line with these findings. Both our and findings from a Norwegian study showed that more women purchased sedative drugs during pregnancy than postpartum. Possibly, some purchases of prescription sedative drugs during pregnancy are likely to have been purchased before awareness of pregnancy explaining the observation that at least partly. We observed that benzodiazepine derivatives anxiolytics or benzodiazepine related drugs were the most commonly purchased prescription sedative drugs similar to previous reports^17,18^. Benzodiazepine derivatives anxiolytics include drugs such as diazepam, oxazepam, and lorazepam and, respectively, benzodiazepine related drugs include drugs such as zopiclone and zolpidem.

In developed countries, 20–90% of pregnant women used prescription drugs; the prevalence being the lowest in the United States and Northern European countries and highest in France and Germany^17,19,20^. Our study findings, 79% of women (non-users of sedative drugs) within 90 days before conception, during pregnancy and/or within 90 days after delivery purchased at least one prescription drugs are in line with these previous study findings. The wide range in the use of prescription drugs is at least in part explained by differences in the prescription versus non-prescription status of some drugs, such as paracetamol, as well as cultural differences in prescribing practices^21,22^. According to previous study findings in pregnant women the most common reasons for the use of prescription drugs are acute respiratory diseases and asthma, alimentary diseases and metabolic disorders, dermatological diseases, nervous system disorders including depression and anxiety, and pain^17,19,21–24^. We found that women most often purchased prescription drugs from the class of anti-infectives for systemic use, musculoskeletal system and nervous system, consistent with previous studies.

Our study findings showed that six out of ten sedative drug users also purchased antidepressants. A Norwegian study showed that in pregnancy two out of ten benzodiazepine or benzodiazepine-related drug users also used antidepressants and according to a U.S study three out of ten, respectively^17,18,25^. Our higher number of users are explained, at least partly, by cultural differences in prescribing drugs and by the fact that our data included prescriptions from all physicians—both private and municipal.

We found no differences in pregnancy outcomes between sedative drug users and non-users except ponderal index of neonates. Previous studies have found that maternal use of benzodiazepine or benzodiazepine-related drugs may be associated with adverse pregnancy outcomes such as preterm birth, increased risk for caesarean sections, low birth weight, small head circumference, low Apgar score, need for neonatal ventilatory support, and need for NICU treatment^14–16,26,27^. Reasons behind the observation on differences in ponderal index between offspring of sedative drug users and non-users during pregnancy remain unclear and larger studies are warranted to confirm the observation.

Our study has several strengths. The study cohort is a complete regional cohort during a seven years follow-up period from the city of Vantaa, Finland, including all primiparous women who fulfilled the criteria. In 2015, the city of Vantaa had about 220,000 inhabitants and about 44,000 women of childbearing age. We used comprehensive, validated, and high-quality register data from Finnish national health registers^28^. In Finland, different registers can be combined using a personal identification number assigned for every citizen since the 1960s. The Social Insurance Institution maintains data on all reimbursements and purchases of prescription drugs in Finland, covering data from primary health care centers, hospitals, and private doctor’s offices. We recognize that there are also weaknesses in our study. We are missing information about indications for use of medication, drug doses, duration of drug use, whether drug use was regular or intermittent, and whether the drug has been used as prescribed. Further, we are missing data on over-the-counter drugs. In our study cohort, the number of women purchasing prescription sedative drugs was small which should be kept on mind when considering the significance of the results. All study participants lived in the city of Vantaa and spoke Finnish or Swedish as native language, thus the generalizability of our study findings is limited.

In conclusion, as expected maternal use of prescription sedative drugs before, during, and after pregnancy was low and the most commonly used prescription drugs were benzodiazepine derivatives anxiolytics. A significant number of primiparous women who used sedative drugs also used antidepressants. Maternal use of prescription sedative drugs does not seem to influence the condition of the offspring during the first week of life.

## Methods

The study cohort consists of all primiparous women, who lived in the city of Vantaa, Finland, and who spoke Finnish or Swedish as native language and who delivered a singleton between January 1st 2009 and December 31st 2015. Vantaa is the fourth most populated city in Finland and it is located in southern Finland in Helsinki metropolitan area. Women were determined as primiparous if they delivered the first time a live birth or a stillbirth from 500 g or 22 gestational weeks onwards. Altogether 6231 primiparous women fulfilled these criteria and composed the study population.

The Finnish Institute for Health and Welfare (THL) maintains the Medical Birth Register to which all Finnish maternity hospitals transmit data on all live births and stillbirths. From this register we obtained the following data of primiparous women and of their pregnancies: age, status of cohabiting, smoking (no smoking, quitting smoking during the first trimester, or smoking during pregnancy), pre-pregnancy weight and height, number of previous pregnancies (induced abortions, miscarriages, and ectopic pregnancies), use on infertility treatments, caesarean sections, and the duration of pregnancy at the day of delivery.

Pre-pregnancy body mass index was the quotient of pre-pregnancy weight (kg) and height (m) squared. The woman was classified as obese, if her body mass index was $\ge$ 30 kg/m^2^. Conception date was calculated by reducing from the delivery date the duration of pregnancy. Birth was classified as preterm birth, if the delivery was before 37 + 0 gestational weeks. Caesarean sections were divided into emergency, urgent, and elective sections.

From Statistics Finland we obtained data on educational attainment: educational attainment was defined according to years of highest attained schooling (http://www.stat.fi/meta/luokitukset/koulutus/001-2016/kuvaus).

From the Social Insurance Institution, we obtained data on chronic diseases three years before conception (http://www.kela.fi/web/en/reimbursements-for-medicine-expenses). For receiving the imbursement, the physician in charge treating the patient composes a medical certificate with a history of disease and status observations of the patient with chronic disease. The expert physicians of the Social Insurance Institution review the medical certificate and if the criteria for the chronic disease is fulfilled, the patient receives a right to a reimbursable medication. At the same time, the entitlement is entered into a nationwide register.

In Finland, people purchased prescription drugs from a pharmacy. The Social Insurance Institution maintains a register of all prescription drug purchases with data on ATC codes and with purchase date. From this source we obtained data on drug purchases with ATC codes and number of prescriptions a person has purchased including purchase date within 90 days before conception, during pregnancy, and within 90 days after delivery. Women who had prescription drug purchases with ATC codes N03AE (clonazepam), N05BA (benzodiazepine derivatives anxiolytics), N05CD (benzodiazepine derivates), or N05CF (benzodiazepine related drugs) within 90 days before conception, during pregnancy, or within 90 days after delivery were considered as users of sedative drugs.

From the Finnish Medical Birth Register we obtained data on offspring sex, birth length and weight, head circumference, Apgar score at 1 min, and need of NICU treatment before age of seven days, need of respiratory treatment, need of resuscitation with intubation, need for antibiotic treatment, and diagnosis of congenital malformations, deformations and chromosomal abnormalities (International Statistical Classification of Diseases and Related Health Problems, 10th revision, [ICD-10] codes Q00-Q99) before the age of seven days. Offspring birthweight was calculated as Z-scores according to sex and gestational age within our own cohort. Ponderal index was calculated dividing the birth weight (kg) by the birth length (m)^3^^29^.

### Ethics approval

The ethics committee of the Hospital District of Helsinki and Uusimaa, Finland (356/13/03/03/2015, November 2, 2015), and the health authority of the city of Vantaa, Finland, have approved the study. Finnish Institute for Health and Welfare (THL), The Finnish Social Insurance Institution and Statistics Finland have given permission to use register data in the study. The study has been conducted in accordance with the Declaration of Helsinki, version 2013. We followed the Strengthening the Reporting of Observational Studies in Epidemiology (STROBE) guidelines in the manuscript preparation and study design.

### Statistical analyses

Data are presented as means with SD, as medians with IQR or as counts with percentages. Statistical comparisons between the groups were done using t-test, and chi-square tests. Hommel’s adjustment has been applied to correct levels of significance for multiple testing at significance level 0.05. The normality of variables was evaluated graphically and using Shapiro–Wilk W test. Stata 16.1 (StataCorp LP; College Station, Texas, USA) statistical package was used for the analysis.

### Consent to participate

Informed consents were not required because this study is a register-based cohort study and none of the study participants were contacted.

## Data Availability

Data cannot be shared for both legal and ethical reasons. Data from the Finnish Institute for Health and Welfare, Statistics Finland, and the Social Insurance Institution can only be used for the purpose stated in the license granted, scientific research on society by the license applicant, and can therefore not be shared with third parties.
